# Effect of prehospital high-dose glucocorticoid on hemodynamics in patients resuscitated from out-of-hospital cardiac arrest: a sub-study of the STEROHCA trial

**DOI:** 10.1186/s13054-024-04808-3

**Published:** 2024-01-22

**Authors:** Laust E. R. Obling, Rasmus P. Beske, Martin A. S. Meyer, Johannes Grand, Sebastian Wiberg, Thomas Mohr, Anders Damm-Hejmdal, Julie L. Forman, Ruth Frikke-Schmidt, Fredrik Folke, Jacob E. Møller, Jesper Kjaergaard, Christian Hassager

**Affiliations:** 1 Department of Cardiology, Rigshospitalet - Copenhagen University Hospital, Blegdamsvej 9, 2100 Copenhagen, OE Denmark; 2 Department of Thoracic Anesthesiology, Rigshospitalet - Copenhagen University Hospital, Copenhagen, Denmark; 3 Department of Intensive Care, Herlev-Gentofte Hospital - Copenhagen University Hospital, Copenhagen, Denmark; 4 Copenhagen Emergency Services, Capital Region of Denmark, Copenhagen, Denmark; 5 Department of Public Health, Section of Biostatistics, University of Copenhagen, Copenhagen, Denmark; 6 Department of Clinical Biochemistry, Rigshospitalet - Copenhagen University Hospital, Copenhagen, Denmark; 7 Department of Clinical Medicine, University of Copenhagen, Copenhagen, Denmark; 8 Department of Cardiology, Herlev-Gentofte Hospital - Copenhagen University Hospital, Copenhagen, Denmark; 9 Department of Cardiology, Odense University Hospital, Odense, Denmark

**Keywords:** Out-of-hospital cardiac arrest, Prehospital intervention, Intensive cardiovascular care, Post-cardiac arrest syndrome, Inflammation, Hemodynamics, Vasopressor

## Abstract

**Background:**

Following resuscitated out-of-hospital cardiac arrest (OHCA), inflammatory markers are significantly elevated and associated with hemodynamic instability and organ dysfunction. Vasopressor support is recommended to maintain a mean arterial pressure (MAP) above 65 mmHg. Glucocorticoids have anti-inflammatory effects and may lower the need for vasopressors. This study aimed to assess the hemodynamic effects of prehospital high-dose glucocorticoid treatment in resuscitated comatose OHCA patients.

**Methods:**

The STEROHCA trial was a randomized, placebo-controlled, phase 2 trial comparing one prehospital injection of methylprednisolone 250 mg with placebo immediately after resuscitated OHCA. In this sub-study, we included patients who remained comatose at admission and survived until intensive care unit (ICU) admission. The primary outcome was cumulated norepinephrine use from ICU admission until 48 h reported as mcg/kg/min. Secondary outcomes included hemodynamic status characterized by MAP, heart rate, vasoactive-inotropic score (VIS), and the VIS/MAP-ratio as well as cardiac function assessed by pulmonary artery catheter measurements. Linear mixed-model analyses were performed to evaluate mean differences between treatment groups at all follow-up times.

**Results:**

A total of 114 comatose OHCA patients were included (glucocorticoid: *n* = 56, placebo: *n* = 58) in the sub-study. There were no differences in outcomes at ICU admission. From the time of ICU admission up to 48 h post-admission, patients in the glucocorticoid group cumulated a lower norepinephrine use (mean difference − 0.04 mcg/kg/min, 95% CI − 0.07 to − 0.01, *p* = 0.02). Moreover, after 12–24 h post-admission, the glucocorticoid group demonstrated a higher MAP with mean differences ranging from 6 to 7 mmHg (95% CIs from 1 to 12), a lower VIS (mean differences from − 4.2 to − 3.8, 95% CIs from − 8.1 to 0.3), and a lower VIS/MAP ratio (mean differences from − 0.10 to − 0.07, 95% CIs from − 0.16 to − 0.01), while there were no major differences in heart rate (mean differences from − 4 to − 3, 95% CIs from − 11 to 3). These treatment differences between groups were also present 30–48 h post-admission but to a smaller extent and with increased statistical uncertainty. No differences were found in pulmonary artery catheter measurements between groups.

**Conclusions:**

Prehospital treatment with high-dose glucocorticoid was associated with reduced norepinephrine use in resuscitated OHCA patients.

*Trial registration*: EudraCT number: 2020-000855-11; submitted March 30, 2020. URL: https://www.clinicaltrials.gov; Unique Identifier: NCT04624776.

**Graphic Abstract:**

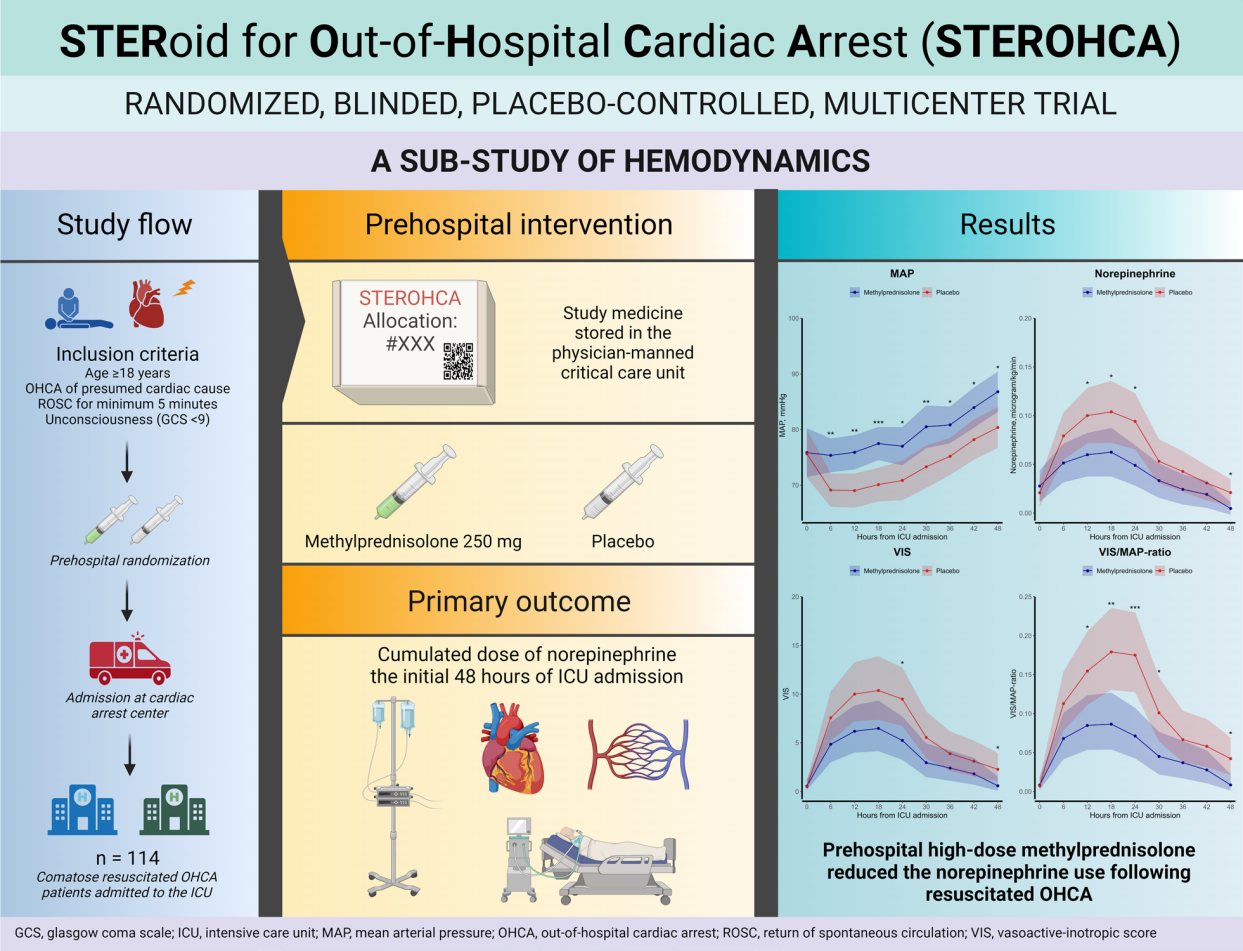

**Supplementary Information:**

The online version contains supplementary material available at 10.1186/s13054-024-04808-3.

## Background

Out-of-hospital cardiac arrest (OHCA) is a critical condition associated with high morbidity and mortality [1]. Upon successful resuscitation, patients who remain unconscious are at a high risk of dying due to the post-cardiac arrest syndrome [2, 3]. During the acute phase, cause of death is most often due to post-resuscitation shock and multiorgan failure, while withdrawal of life support due to neurological damage is the main driver of death later on following resuscitation [4, 5]. Vasopressors are recommended to maintain mean arterial pressure (MAP) above 65 mmHg, but excessive vasopressor use, and a continuously low MAP are both associated with poor outcomes in OHCA [6, 7]. Myocardial dysfunction and ischemic/reperfusion injury involving systemic inflammation play central roles in clinical deterioration of the patient in the initial days after admission [8, 9].

Glucocorticoids have previously been associated with faster shock resolution and reduced need for vasopressors [10, 11]. Whether this also applies in post-cardiac arrest treatment remains to be proven.

To explore the possible advantages of glucocorticoids in post-cardiac arrest treatment, we conducted the “STERoid treatment as anti-inflammatory and neuroprotective agent following OHCA” (STEROHCA) trial [12]. Here, following a median time of twenty minutes from return of spontaneous circulation (ROSC), one high-dose glucocorticoid injection was administered prehospitally in resuscitated OHCA patients in order to mitigate severity of the post-cardiac arrest syndrome. The intervention showed a marked anti-inflammatory effect without affecting markers of neurological damage [13].

The present STEROHCA sub-study aims to assess how early anti-inflammatory treatment with high-dose glucocorticoid affects hemodynamics and the use of vasopressor treatment in the post-resuscitation phase following OHCA.

## Methods

### Trial design

This was a sub-study of the STEROHCA trial, an investigator-initiated randomized placebo-controlled multicenter study investigating the anti-inflammatory and neuroprotective effects of early high-dose glucocorticoid treatment following resuscitated OHCA. The study protocol and the primary results have previously been published [12]. Prior to initiation, approvals for the study were obtained from the Regional Ethics Committee (ID: H-20022320) and the Danish Medicines Agency (ID: 2020033425). Further, the trial was registered at clinicaltrials.gov (NCT04624776), and monitored for Good Clinical Practice. According to the Declaration of Helsinki and national requirements, informed consent was provided from an independent physician prior to inclusion, from relatives after hospital admission, and from patients surviving and deemed cognitive habile to understand information regarding the study.

### Patients

The study was performed at two cardiac arrest centers, Rigshospitalet and Gentofte Hospital, and the Emergency Medical Services, Capital Region, Denmark. From October 2020 to July 2022, 158 patients were enrolled. Of these, 137 patients encompassed the modified intention-to-treat population. Patients resuscitated from OHCA were prehospitally enrolled according to the following inclusion criteria: Age ≥ 18 years, unconsciousness (Glasgow Coma Scale ≤ 8), OHCA due to a presumed cardiac cause, and minimum five minutes of ROSC. In brief, the main exclusion criteria were asystole as primary rhythm and known therapy limitation. All inclusion and exclusion criteria are provided in the Additional file 1: Appendix. In the present sub-study, only patients who arrived at the hospital in a comatose state and were subsequently admitted to an ICU were studied.

### Randomization

A random number generator was used to create the allocation sequence, randomizing patients in a 1:1 ratio in permuted blocks of four. Study medication and placebo were placed in indistinguishable opaque boxes, assigned with random numbering according to allocation. Subsequently from inclusion of a patient, the prehospital staff were unblinded upon opening of a study box and administering study medication. In-hospital personnel, in addition to patients, relatives and outcome assessors, remained blinded for allocation.

### Intervention

Patients were assigned to receive either a bolus injection of 250 mg methylprednisolone (Solu-Medrol, Pfizer©) or placebo in the prehospital setting. Study medication was administered intravenously or intraosseous at the discretion of the treating physician. The intervention was completed as soon as possible following resuscitation with obtained ROSC for a minimum of 5 min and always before hospital arrival.

### Concomitant care

All patients underwent conventional treatment according to international post-resuscitation guidelines [6]. This included maintenance of a targeted temperature of 36° C in comatose patients, sedation primarily with propofol and fentanyl, and using vasopressors and inotropes at the discretion of the treating physician. Neurologic prognostication followed contemporary guidelines and remained blinded to the treatment. Balloon-tipped pulmonary artery catheters (PAC, Edwards Lifesciences, Irvine, CA) were routinely inserted at one of the sites (Rigshospitalet), but not at the other. PACs were inserted through the internal jugular or subclavian vein and subsequently removed either at the time of discharge from the ICU or after 72 h unless it was required for further clinical hemodynamic monitoring.

### Objectives and outcome assessments

The aim of this sub-study was primarily to assess how high-dose glucocorticoid affected vasopressor use in the acute hospitalization phase following resuscitated cardiac arrest. Norepinephrine was the first-line vasopressor used at the two sites, and we defined the acute phase as the initial 48 h of ICU admission. The primary outcome was the average dose of norepinephrine used from ICU admission to 48 h in each patient. Secondarily, to further characterize hemodynamics and pharmacological supportive treatment, we examined MAP, heart rate and the vasoactive-inotropic score (VIS) which is calculated by all vasoactive and inotropic medications administered, reflecting support of the cardiovascular system [14]. A VIS/MAP-ratio was calculated at all time points to quantify the relationship between vasopressor and inotropic support provided and MAP, with a lower ratio indicating less reliance on pharmacological support to reach a certain MAP target, and a higher ratio suggesting the need for more aggressive treatment. We assessed central venous pressure (CVP), mean pulmonary arterial pressure (PAPm), cardiac output (CO), pulmonary capillary wedge pressure (PCWP), systemic vascular resistance (SVR), and mixed venous saturation (SvO2). Thermodilution-based CO, central venous blood for SvO2 drawn from the PAC, PCWP, and SVR measurements were only available in patients from one of the sites (Rigshospitalet), where PACs were routinely implanted. As secondary outcomes, we also aimed to assess clinical status and myocardial injury. Overall sequential organ failure assessment (SOFA) score and cardiovascular SOFA alone were assessed on calendar days 1–3 to describe clinical status. Further, survival status at hospital discharge and a minimum of 180 days from the cardiac arrest were obtained. Myocardial injury was characterized by the trajectory of Troponin T (TnT) or Troponin I (TnI) depending on the site, Creatine Kinase MB (CKMB), and N-terminal pro B-type natriuretic peptide (NT-proBNP). Biomarker measurements were performed using a COBAS 8000 and analyzed as part of routine biochemistry using a DS/EN ISO 15189 standardized laboratory.

### Statistical methods

All analyses in this sub-study were conducted in comatose patients within the modified intention-to-treat population from the STEROHCA trial. Dichotomous outcomes were presented with counts (n) and percentages (%) and compared between treatment groups using the Chi-squared test or Fisher exact test. Continuous outcomes were presented with medians and quartiles and compared with a Mann–Whitney U test. The primary outcome was compared between treatment groups using a two-sample t-test. Moreover, a sensitivity analysis was performed using a linear regression model to examine potential interactions between the treatment group and temperature target (specifically, 36 degrees Celsius vs. fever avoidance). Temporal differences between groups were compared using a linear mixed model including time, treatment, and the treatment-by-time interactions as fixed effects and with an unstructured covariance pattern to account for repeated measurements on each study participant. Skewed outcomes were log-transformed prior to analysis to approximate normal distribution (TnT, TnI, CKMB, and NT-proBNP) or square root function if values of “0” were present (norepinephrine, VIS, and VIS/MAP-ratio). Comparisons were made at all times of measurement predefined in the study protocol and reported as mean difference with 95% confidence interval (CI). The results are further visualized as group-specific medians with CI after back-transformation if necessary. Missing data were handled implicitly via maximum likelihood estimation in the linear mixed models.

All analyses were conducted with R version 4.2.2 [15]. The LMMstar-package [16] was used for linear mixed model analyses. Statistical significance was defined as a *p* value below 0.05 for all analyses.

## Results

**Fig. 1:**
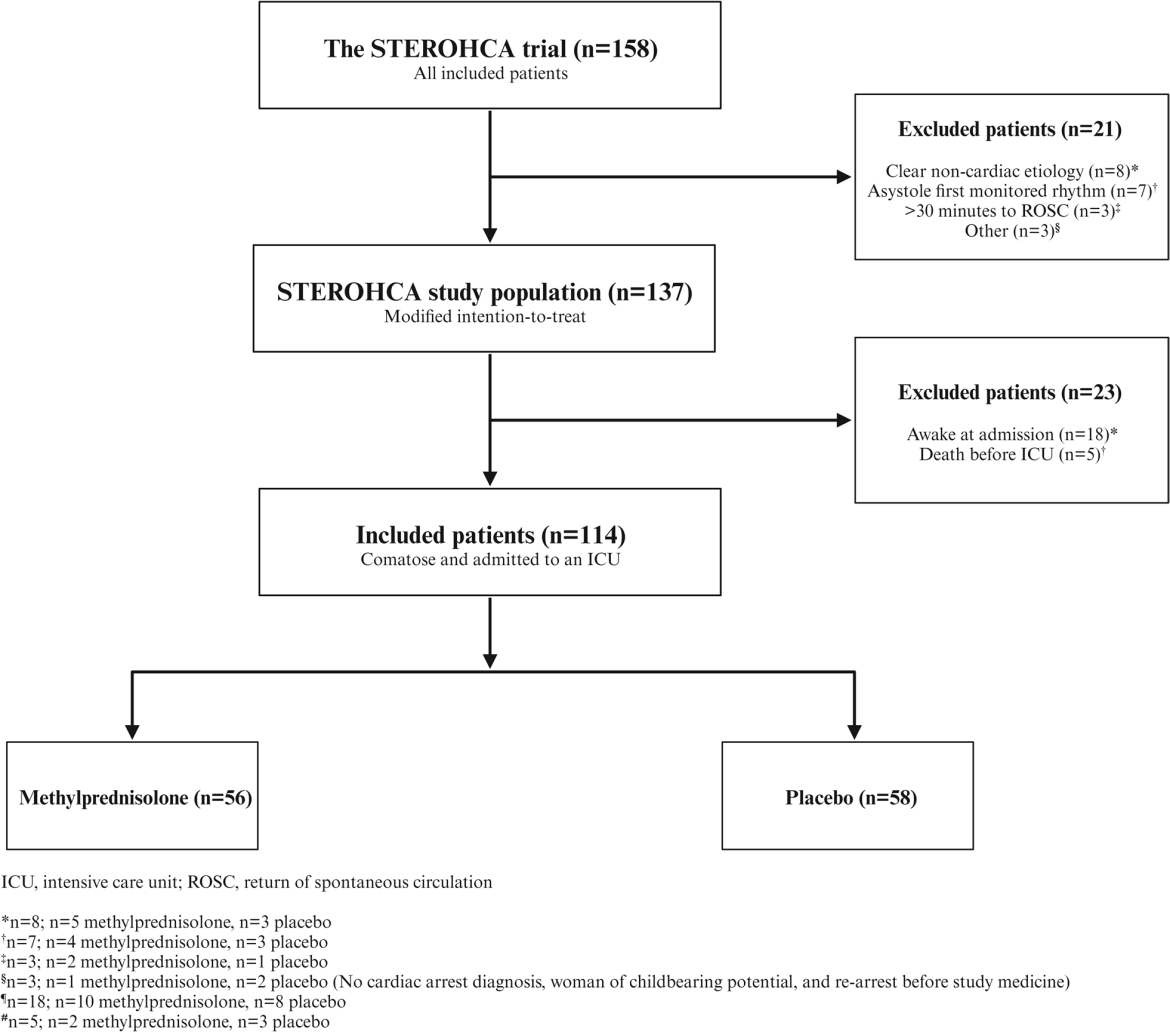
Consort flow diagram

A total of 137 patients encompassed the modified intention-to-treat population in the STEROHCA trial. In the present sub-study, we only included comatose patients who were admitted to an ICU, resulting in 114 patients allocated to glucocorticoid (*n* = 56) or placebo (*n* = 58). Hence, 18 awake patients were not included (*n* = 10 glucocorticoid, *n* = 8 placebo), and 5 patients died before admission to the ICU (*n* = 2 glucocorticoid, *n* = 3 placebo). The study flowchart can be seen in Fig. 1. The median age was 67 years (57, 74), and the 180-day survival was 73% (*n* = 41/56) and 62% (*n* = 36/58) in the glucocorticoid and placebo group, respectively. Neurological outcome after 180 days is visualized in Additional file 2: Fig. S1. In the glucocorticoid group, a greater number of patients received amiodarone during resuscitation when compared to the placebo group (*n* = 27 (48%) vs. *n* = 14 (24%), *p* = 0.007). During the initial 48 h of ICU admission 8 patients died (*n* = 2 glucocorticoid, *n* = 6 placebo). The body temperature within the initial 24 h of TTM showed no difference between treatment groups. A list of adverse events, including serious adverse events, can be seen in Additional file 2: Table S1*.* Patient characteristics are summarized in Table 1.

**Table 1:** Patient characteristics before- and after inclusion

	Treatment group		
	Placebo, *N* = 58	Methylprednisolone, *N* = 56	*p* value
**Before inclusion**			
Demographic characteristics			
Age, years, median (IQR)	67 (57, 75)	66 (58, 72)	0.7
Male, *n* (%)	45 (78%)	45 (80%)	0.7
Ischemic heart disease, *n* (%)	15 (26%)	9 (16%)	0.2
Heart failure, *n* (%)	10 (17%)	14 (25%)	0.4
Atrial fibrillation, *n* (%)	11 (19%)	7 (12%)	0.4
Prehospital variables			
Witnessed arrest, *n* (%)	53 (91%)	45 (80%)	0.09
Bystander CPR, *n* (%)	49 (84%)	50 (89%)	0.4
Epinephrine administered, *n* (%)	28 (48%)	39 (70%)	0.02
Amiodarone administered, *n* (%)	14 (24%)	27 (48%)	0.007
Time to ROSC, min, median (IQR)	14 (10, 20)	18 (15, 21)	0.02
Post-resuscitation ECG rhythm, n (%)			0.4
*Sinus rhythm*	44 (76%)	40 (71%)	
*Atrial fibrillation*	12 (21%)	10 (18%)	
Other*	2 (3%)	6 (11%)	
Post-resuscitation ECG, signs of ischemia, *n* (%)			0.2
*STEMI*	24 (41%)	24 (43%)	
*LBBB or RBBB*	19 (33%)	15 (27%)	
*Unspecific ischemia†*	7 (12%)	2 (4%)	
*No ischemia*	8 (14%)	15 (27%)	
**After inclusion, in-hospital**			
Hospital arrival characteristics			
LVEF at arrival, (%), median (IQR)	38 (25, 45)	40 (29, 50)	0.6
Lactate at arrival, mmol/L, median (IQR)	4.6 (2.8, 6.8)	5.3 (3.6, 6.8)	0.3
Cardiogenic shock at arrival, *n* (%)	6 (10%)	2 (4%)	0.3
Acute CAG, *n* (%)	38 (66%)	31 (55%)	0.3
Acute PCI, *n* (%)	22 (58%)	18 (60%)	0.9
During admission			
ICU length of stay, days, median (IQR)	5.1 (2.6, 7.6)	4.1 (2.1, 6.5)	0.5
Ventilator, days, median (IQR)	2.7 (1.3, 4.3)	1.9 (1.0, 3.5)	0.2
Best LVEF in the ICU, (%), median (IQR)	40 (30, 50)	45 (35, 50)	0.4
Day 1, SOFA cardiovascular score, median (IQR)	4.0 (3.0, 4.0)	4.0 (3.0, 4.0)	0.2
Day 2, SOFA cardiovascular score, median (IQR)	4.0 (3.0, 4.0)	3.0 (3.0, 4.0)	0.01
Day 3, SOFA cardiovascular score, median (IQR)	3.0 (0.0, 3.3)	0.0 (0.0, 3.0)	0.009
Day 1, SOFA total score, median (IQR)	12.0 (11.0, 13.0)	12.0 (11.0, 13.0)	0.6
Day 2, SOFA total score, median (IQR)	11.5 (8.8, 12.3)	11.0 (9.5, 12.0)	0.3
Day 3, SOFA total score, median (IQR)	8.0 (5.2, 11.0)	7.5 (4.2, 10.0)	0.3
Death before hospital discharge, *n* (%)	21 (36%)	14 (25%)	0.2
Death from any cause at 180 days, *n* (%)	22 (38%)	15 (27%)	0.2

*CAG* coronary angiography, *CPR* cardiopulmonary resuscitation, *ECG*, electrocardiogram, *ICU* intensive care unit, *IQR* interquartile range, *LBBB* left bundle branch block, *LVEF* left ventricular ejection fraction, *PCI* percutaneous coronary intervention, *RBBB* right bundle branch block, *ROSC* return of spontaneous circulation, *SOFA* sequential organ failure assessment, *STEMI* ST-elevation myocardial infarction

*Including pace rhythm, nodal rhythm, and sinus bradycardia

^†^Non-specific ST-segment depression/elevation or T-wave changes

### Hemodynamic parameters

**Fig. 2:**
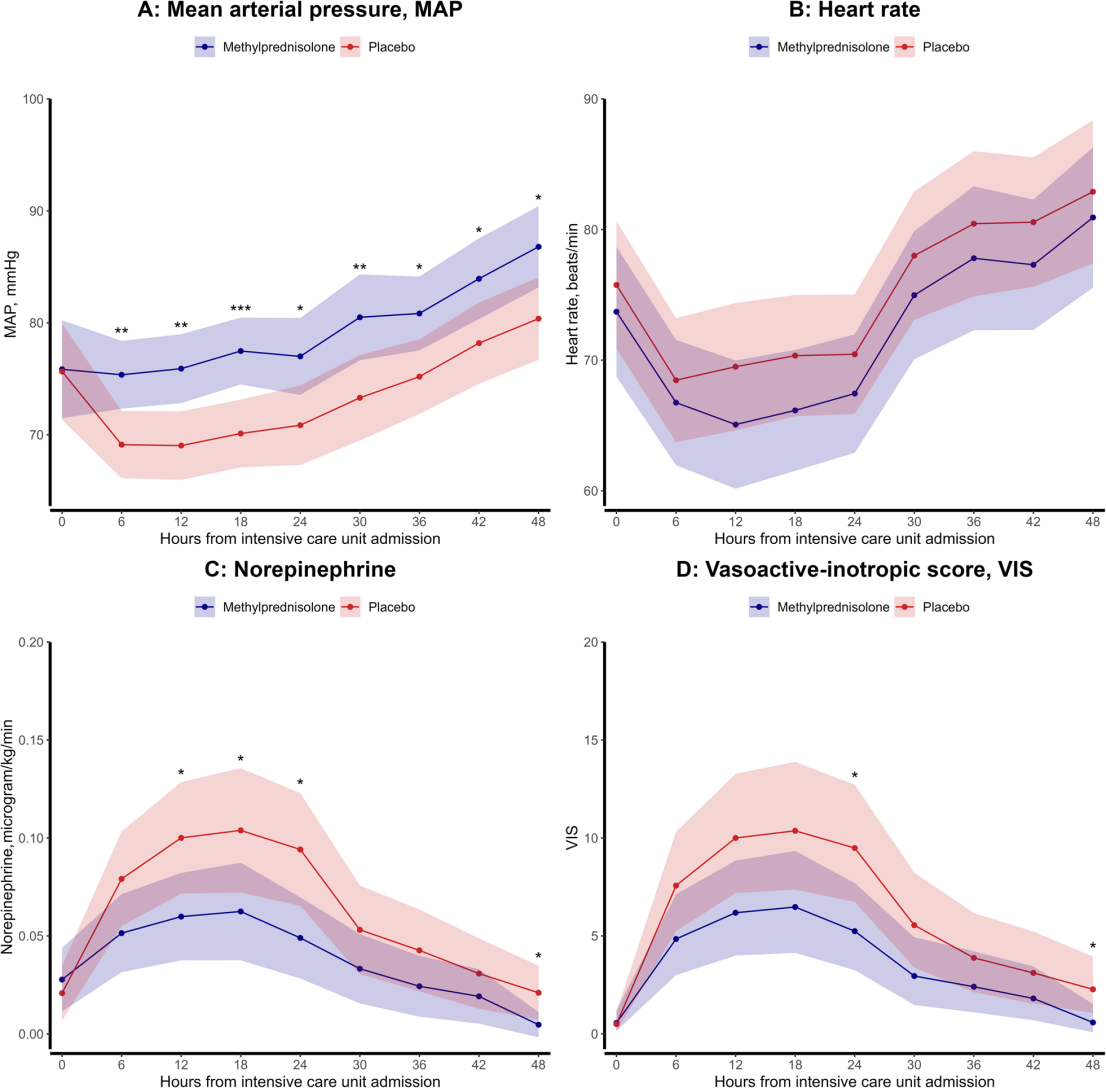
**A**–**D** Hemodynamic assessments; **A** mean arterial pressure (mmHg) according to randomization, **B** heart rate (beats/minute) according to randomization, **C** norepinephrine use (mcg/kg/min) according to randomization, **D** vasoactive-inotropic score according to randomization. Each variable is depicted as estimated marginal means with 95% confidence intervals to each time point to demonstrate differences between treatment groups. If a *p* value is < 0.05, it is marked with one star (*), if a *p* value is < 0.01, it is marked with two stars (**), and if a *p* value is < 0.001, it is marked with three stars (***). The figure includes the measurements for all patients included in the sub-study (*n* = 114)

The glucocorticoid group showed a lower cumulative dose of norepinephrine from ICU admission to 48 h after (mean difference − 0.04 mcg/kg/min, 95% CI − 0.07 to − 0.01, p = 0.02). There was no interaction of treatment group and temperature target on norepinephrine use (Treatment group x temperature target, *p* = 0.55). Although the norepinephrine dose was similar at ICU admission between the groups [0.03 mcg/kg/min (95% CI 0.01–0.04) vs. 0.02 mcg/kg/min (95% CI 0.01–0.03), *p* = 0.64], the glucocorticoid group exhibited lower norepinephrine use at various time points, with the most significant difference observed after 24 h (0.05 mcg/kg/min (95% CI 0.03–0.07) vs. 0.09 mcg/kg/min (95% CI 0.07–0.12), *p* = 0.01). No difference in MAP was found between the two groups at ICU admission (76 mmHg (95% CI 72–80) vs. 76 mmHg (95% CI 71–80), *p* = 0.95). However, from 6 to 48 h, the glucocorticoid group consistently exhibited higher MAP values, with the most significant difference noted after 18 h (78 mmHg (95% CI 74–81) vs. 70 mmHg (95% CI 67–74), *p* < 0.001). Consequently, from ICU admission to 48 h, the glucocorticoid group demonstrated lower values of the VIS at various time points. No differences in heart rate were observed between the two groups. The treatment effect for the hemodynamic parameters is depicted in Fig. 2A–D.

**Fig. 3:**
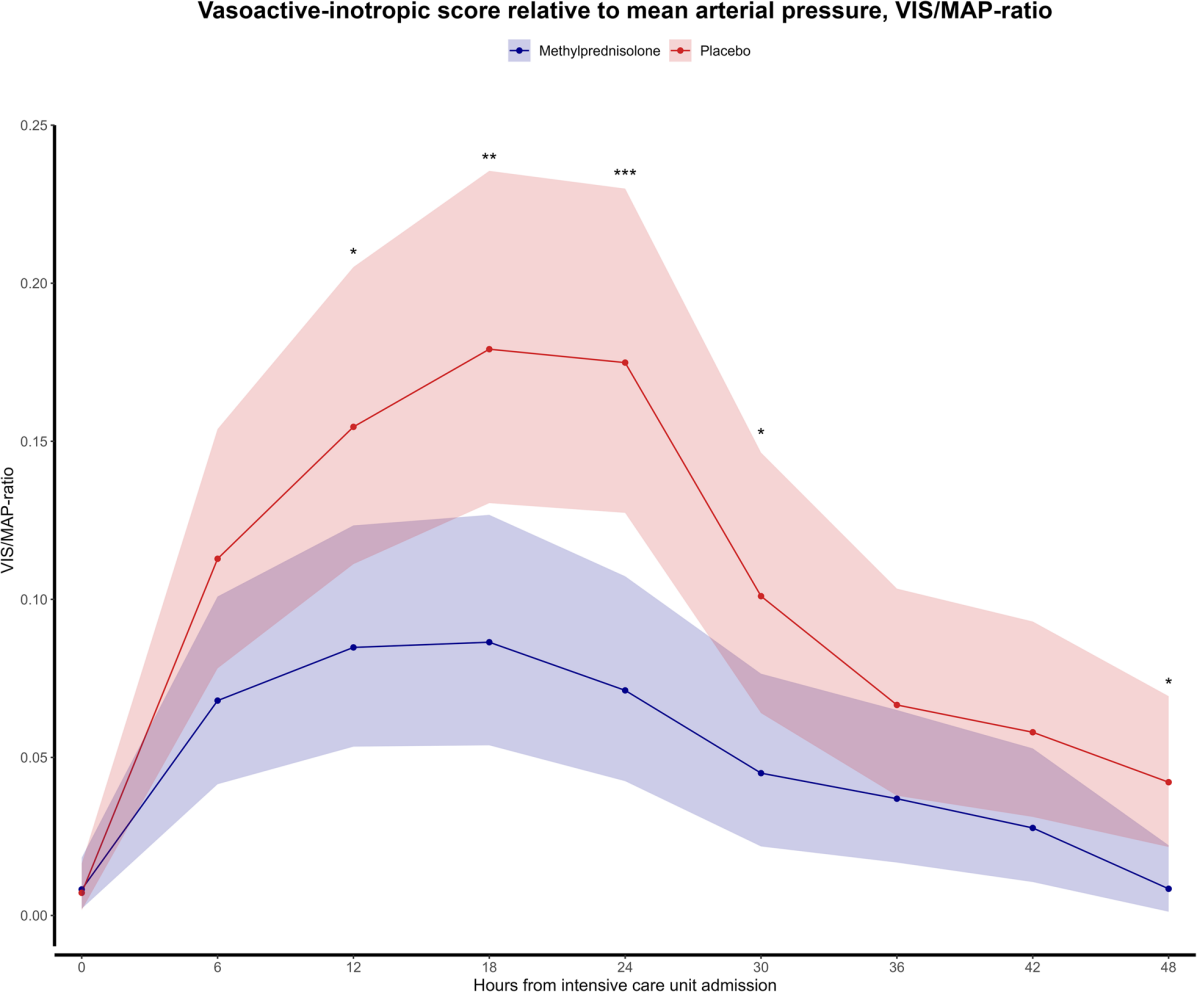
Vasoactive-inotropic score/mean arterial pressure-ratio, defining the cardiovascular response to treatment with vasopressors and inotropes, according to randomization, depicted as estimated marginal means with 95% confidence intervals to each time point to demonstrate differences between treatment groups. If a *p* value is < 0.05, it is marked with one star (*), if a *p* value is < 0.01, it is marked with two stars (**), and if a *p* value is < 0.001, it is marked with three stars (***). The figure includes the measurements for all patients included in the sub-study (*n* = 114)

Evaluation of the VIS/MAP ratio revealed lower ratios in the glucocorticoid group at several time points, as illustrated in Fig. 3.

Hemodynamic values, along with their corresponding confidence intervals at each time point, are summarized in Additional file 2: Table S2.

**Fig. 4:**
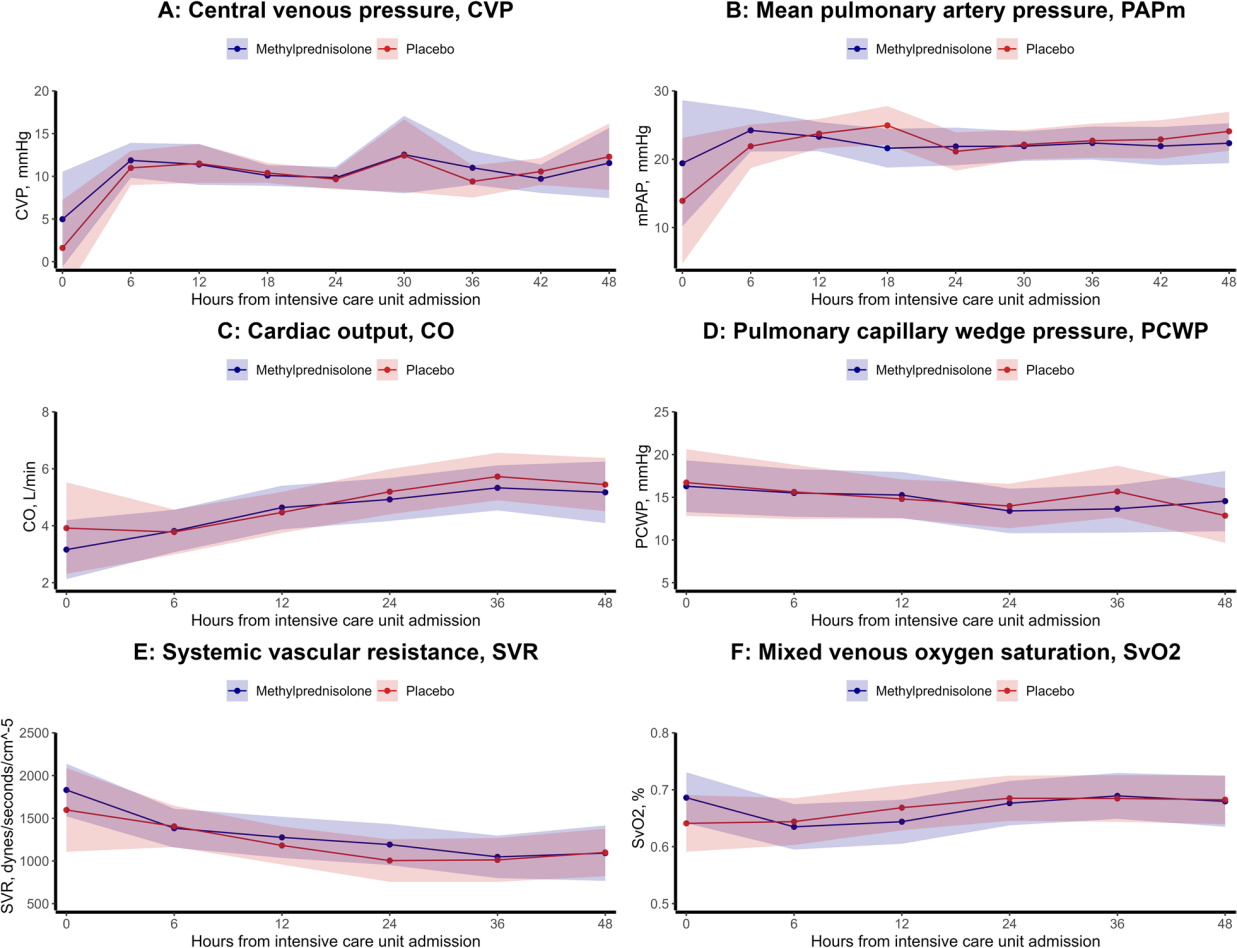
**A**–**F** Cardiac function assessments; **A** central venous pressure (mmHg) according to randomization, **B** mean pulmonary arterial pressure (mmHg) according to randomization, **C** cardiac output (mcg/kg/min) according to randomization, **D** pulmonary capillary wedge pressure (mmHg) according to randomization, **E** systemic vascular resistance (dynes/seconds/cm^−5^) according to randomization, **F** mixed venous oxygen saturation (%) according to randomization. Each variable is depicted as estimated marginal means with 95% confidence intervals to demonstrate differences between treatment groups. The figure includes the central venous pressure and mean pulmonary arterial pressure measurements for all patients included in the sub-study (*n* = 114), while only patients with a pulmonary artery catheter had cardiac output, pulmonary capillary wedge pressure, systemic vascular resistance, and mixed venous oxygen saturation measurements (*n* = 54)

At Rigshospitalet, a PAC was inserted in 54 (68%) patients as soon as possible after admission, and all patients had at least one measurement performed during ICU stay or until dying. Baseline characteristics of patients with- and without a PAC inserted are summarized in Additional file 2: Table S3. No differences in CVP, PAPm, SvO2, SVR, PCWP or CO were found between the two treatment arms, Fig. 4A–F.

### Clinical parameters

No distinctions between groups were observed in ICU length of stay, ventilator days, or SOFA total score during the initial three days of admission. Nevertheless, on the second and third day, the glucocorticoid group exhibited a reduction in SOFA cardiovascular score. There was no difference in mortality rates at hospital discharge or 180 days after the cardiac arrest. All clinical outcomes are summarized in Table 1*.*

### Myocardial injury

There was no difference between groups in biomarkers of myocardial injury or NT-proBNP during the initial 48 h of admission, Additional file 2: Fig. S2.

## Discussion

This was a sub-study of the randomized STEROHCA trial, investigating a single high-dose glucocorticoid injection in the prehospital setting after OHCA. The objective of the sub-study was to evaluate the hemodynamic effects of this early and potent anti-inflammatory treatment. The intervention resulted in a decrease in vasopressor use necessary to achieve a specific MAP.

Glucocorticoids have abundant anti-inflammatory properties and thereby may improve patient outcomes following OHCA [17]. Norepinephrine is often a first-line vasoactive agent used in the management of hemodynamic instability following cardiac arrest [6]. In accordance with previous data [18], our study suggests that the administration of high-dose glucocorticoids may contribute to improved vascular responsiveness, thereby reducing the need for exogenous catecholamines. Likewise, Meyer et al. observed a decrease in VIS during the post-resuscitation phase after administration of the interleukin 6 antagonist Tocilizumab [19]. This reduction in norepinephrine use may be of importance, as high-dose vasopressors have been associated with adverse outcomes, including impaired microvascular function and death [20, 21]. Further, the lower VIS values and VIS/MAP ratios in the glucocorticoid group suggest that patients receiving high-dose glucocorticoids generally had improved hemodynamics and required less intensive vasopressor and inotropic support. These findings underscore the potential for glucocorticoids to mitigate the systemic inflammatory response and improve the vasoplegic mechanisms observed after cardiac arrest. In the STEROHCA trial, a solitary initial high-dose of glucocorticoid was administered to assess the effectiveness in alleviating the inflammatory response. Meanwhile, an ongoing national Danish trial is exploring the potential benefits of maintaining high-dose dexamethasone treatment during the acute phase following admission on clinical outcome (NCT05895838).

Interestingly, the glucocorticoid group exhibited higher MAP compared to the placebo group. Adequate perfusion pressure is critical for ensuring organ perfusion and oxygen delivery in post-cardiac arrest patients, and former studies have found that exposure to hypotension is associated with a higher mortality following OHCA [7, 22]. A study by Adrie et al. [23] found that the immunoinflammatory response in post-resuscitation cardiac arrest patients was comparable with that of sepsis, and treatment with glucocorticoids in septic shock models have demonstrated faster shock resolution and reduced reliance on vasopressors [10, 24], however, no disparities in clinical outcomes have been identified. This could vary in the current study, as the clinical attributes of PCAS diverge from the infection-driven pathophysiology observed in sepsis and septic shock, and further because the intervention was performed at an early crucial point before deterioration of the patients. In the present study, the elevated MAP in the glucocorticoid group could potentially reduce the risk of ischemic injury, although no difference was found in CO and SvO2. The mechanistic effects may be explained by post-resuscitation adrenal insufficiency which induces hypotension and lower effectiveness of vasopressors [25, 26]. The current sub-study indicates that the same effects observed previously after treatment with high-dose glucocorticoids in septic shock, may also adhere to early prehospital administration after OHCA.

In line with the modified intention-to-treat population in the STEROHCA trial, the glucocorticoid group displayed a longer median time to ROSC and a higher frequency of receiving epinephrine and amiodarone during resuscitation and thus before randomization. These factors were present prior to the intervention, and the skewed distribution observed between the groups, is a potential challenge in clinical trials with a limited number of patients. In this context, it accentuates the fact that, the glucocorticoid group by chance may have been admitted in a more compromised state, characterized by unfavorable prehospital prognostic characteristics, including receiving more amiodarone, which is known to cause hypotension [27].

Myocardial ischemia during cardiac arrest leads to impaired contractility, which is then aggravated through ischemic/reperfusion injury due to an excessive cascade of pro-inflammatory cytokines and elevated circulating catecholamines [9]. Systemic inflammation contributes to myocardial injury and further to multiorgan dysfunction complicating the clinical status of post-cardiac arrest patients [28, 29]. In recent years, two animal studies have found that high-dose glucocorticoid treatment reduced myocardial dysfunction and improved microcirculation in the post-resuscitation phase [30, 31]. These results are encouraging, but in the present study, factors of myocardial function, CO and PCWP, were similar between the two groups. This supports that the hemodynamic effects we observed were solely driven by mitigation of vasoplegia and possibly a higher affinity for vasopressors, which is in line with previous observations of early vasoplegic mechanisms following resuscitated OHCA [32]. These early effects may indicate that the pathophysiologic pathways of PCAS have more in common with trauma than the infection-driven response seen in sepsis. Further, despite the anticipation of discovering variations in SVR between the treatment groups based on these results, our observations did not reveal such differences, which may be attributed to insufficient statistical power. Our findings also raise the questions, whether the dose of glucocorticoid administered in the present trial was high enough to ameliorate cardiac function in post-cardiac arrest patients and if continuous in-hospital administration of glucocorticoid could have further improved the clinical status of the patients. Additionally, the current trial did not identify any cardioprotective effects as indicated by biomarkers. However, this sub-study was not designed to address this outcome, and we anticipate that an ongoing trial from our institution will provide further insights into this matter, although only including patients with ST-elevation myocardial infarction (NCT05462730).

Two prior studies focusing on in-hospital cardiac arrest both failed to demonstrate significant hemodynamic benefits following high-dose glucocorticoid administration [33, 34]. However, the outcomes from this study may diverge from the present study for several reasons. Firstly, patients suffering from OHCA, typically have less comorbidity compared to in-hospital cardiac arrest patients, potentially influencing their response to treatment [35]. Secondly, in this study high-dose glucocorticoid was administered at a critical juncture, prior to the onset of a widespread inflammatory response in the patients. In contrast, previous trials employed a lower dose during resuscitation, with additional supportive doses administered after resuscitation in one of those trials. These distinct factors underscore the importance of recognizing that the dynamics and outcomes in OHCA cases may significantly differ from in-hospital scenarios, thereby warranting a fresh perspective on the role of glucocorticoid treatment in such settings.

This sub-study has limitations. The analyses were primarily post-hoc, and not described in the original study protocol, so the results can only be considered as hypothesis generating. Temperature target management could confound the results, however, there was no difference in temperature between treatment groups the initial 24 h of admission. The generalizability is limited, due to the small geographical size of the Capital Region of Denmark, and the high proportion of bystander responders.

## Conclusions

In conclusion, the administration of high-dose glucocorticoid immediately after ROSC leads to reduced norepinephrine use in the post-resuscitation phase after OHCA.

### Supplementary Information

**Additional file 1.** Supplementary appendix.

**Additional file 2.** Supplementary tables and figures.

## Data Availability

Upon a reasonable request, the data substantiating the conclusions of this study can be obtained from the corresponding author (LERO).

## Abbreviations

CI: Confidence interval
CKMB: Creatine kinase MB
CO: Cardiac output
CPC: Cerebral performance category
CVP: Central venous pressure
EDTA: Ethylenediaminetetraacetic acid
hsCRP: High-sensitivity C-reactive protein
ICU: Intensive care unit
MAP: Mean arterial pressure
NT-proBNP: N-Terminal pro B-type natriuretic peptide
OHCA: Out-of-hospital cardiac arrest
PAC: Pulmonary artery catheter
PAPm: Mean pulmonary arterial pressure
PCAS: Post-cardiac arrest syndrome
PCWP: Pulmonary capillary wedge pressure
ROSC: Return of spontaneous circulation
SOFA: Sequential organ failure assessment
SvO2: Mixed venous saturation
SVR: Systemic vascular resistance
TnT: Troponin T
TnI: Troponin I
VIS: Vasoactive-inotropic score
