# Supplementary material for: Effect of prehospital high-dose glucocorticoid on hemodynamics in patients resuscitated from out-of-hospital cardiac arrest: a sub-study of the STEROHCA trial
Source: Crit Care. 2024 Jan 22;28:28. doi: 10.1186/s13054-024-04808-3 (PMC10801994; doi:10.1186/s13054-024-04808-3)
Supplement: Supplementary file 1 — Additional file 1. Supplementary appendix. [file 13054_2024_4808_MOESM1_ESM.docx]

**Supplementary Appendix**

**Inclusion criteria for the STEROHCA trial:**

1. Age ≥ 18 years

2. OHCA of presumed cardiac cause

3. Unconsciousness (GCS ≤ 8) upon pre-hospital randomization

4. Sustained ROSC for at least 5 min

5. Randomization and start of study medicine infusion within 30 minutes of sustained ROSC

**Exclusion criteria for the STEROHCA trial:**

1. Advanced life support termination-of-resuscitation (TOR) exclusion criteria

2. Asystole as primary electrocardiogram (ECG) rhythm

3. Women of childbearing potential

4. Known therapy limitation (known decision made of no resuscitation or intensive therapy)

5. Known allergy to methylprednisolone

6. Known pre-arrest modified Rankin Scale (mRS) score of 4–5

7. Temperature upon randomization < 30° C 8. > 30 min to sustained ROSC
