# Supplementary material for: Effect of prehospital high-dose glucocorticoid on hemodynamics in patients resuscitated from out-of-hospital cardiac arrest: a sub-study of the STEROHCA trial
Source: Crit Care. 2024 Jan 22;28:28. doi: 10.1186/s13054-024-04808-3 (PMC10801994; doi:10.1186/s13054-024-04808-3)
Supplement: Supplementary file 2 — Additional file 2. Supplementary tables and figures. [file 13054_2024_4808_MOESM2_ESM.docx]

**Supplementary table 1. Adverse events**

|  | **Treatment group** | |  |
| --- | --- | --- | --- |
|  | **Placebo**, N = 58 | **Methylprednisolone**, N = 56 | **p-value** |
| AE, patients with ≥1, no. (%) | 45 (78%) | 50 (89%) | 0.09 |
| AE infection, no. (%) | 8 (14%) | 6 (11%) | 0.6 |
| AE bleeding, no. (%) | 6 (10%) | 1 (1.8%) | 0.11 |
| AE dialysis, no. (%) | 2 (3.4%) | 2 (3.6%) | >0.9 |
| AE electrolyte, no. (%) | 15 (26%) | 26 (46%) | 0.02 |
| AE metabolic, no. (%) | 7 (12%) | 25 (45%) | <0.001 |
| AE arrhythmia, no. (%) | 15 (26%) | 11 (20%) | 0.4 |
| AE seizures, no. (%) | 14 (24%) | 11 (20%) | 0.6 |
| SAE, patients with ≥1, no. (%) | 32 (55%) | 29 (52%) | 0.7 |
| SAE infection, no. (%) | 4 (6.9%) | 4 (7.1%) | >0.9 |
| SAE bleeding, no. (%) | 3 (5.2%) | 0 (0%) | 0.2 |
| SAE dialysis, no. (%) | 2 (3.4%) | 2 (3.6%) | >0.9 |
| SAE electrolyte, no. (%) | 1 (1.7%) | 2 (3.6%) | 0.6 |
| SAE metabolic, no. (%) | 0 (0%) | 1 (1.8%) | 0.5 |
| SAE arrhythmia, no. (%) | 9 (16%) | 5 (8.9%) | 0.3 |
| SAE seizures, no. (%) | 13 (22%) | 11 (20%) | 0.7 |
| AE, adverse event; SAE, serious adverse event | | | |

**Supplementary table 2. Hemodynamic variables, estimated marginal means according to treatment group and the between group difference at each time point**

| **Treatment group** | **T0** | **T6** | **T12** | **T18** | **T24** | **T30** | **T36** | **T42** | **T48** |
| --- | --- | --- | --- | --- | --- | --- | --- | --- | --- |
| **Norepinephrine, mcg/kg/min** |  |  |  |  |  |  |  |  |  |
| Glucocorticoid | 0.03 (0.01, 0.04) | 0.05 (0.03, 0.07) | 0.06 (0.04, 0.08) | 0.06 (0.04, 0.09) | 0.05 (0.03, 0.07) | 0.03 (0.02, 0.05) | 0.02 (0.009, 0.04) | 0.02 (0.005, 0.03) | 0.005 (-0.001, 0.01) |
| Placebo | 0.02 (0.01, 0.03) | 0.08 (0.06, 0.10) | 0.10 (0.07, 0.13) | 0.10 (0.07, 0.14) | 0.09 (0.07, 0.12) | 0.05 (0.03, 0.08) | 0.04 (0.02, 0.06) | 0.03 (0.01, 0.05) | 0.02 (0.007, 0.03) |
| *Between group difference* | 0.01 (-0.01, 0.03) | -0.03 (-0.06, 0.004) | -0.04 (-0.08, -0.004) | -0.04 (-0.08, -0.001) | -0.05 (-0.08, -0.009) | -0.02 (-0.05, 0.009) | -0.02 (-0.04, 0.008) | -0.01 (-0.03, 0.01) | -0.02 (-0.03, -0.001) |
| **MAP, mmHg** |  |  |  |  |  |  |  |  |  |
| Glucocorticoid | 76 (73, 79) | 75 (72, 79) | 76 (73, 79) | 78 (74, 81) | 77 (74, 80) | 80 (77, 84) | 81 (77, 84) | 84 (81, 88) | 87 (83, 90) |
| Placebo | 76 (72, 79) | 69 (66, 73) | 69 (66, 73) | 70 (67, 74) | 71 (68, 75) | 75 (71, 78) | 76 (73, 80) | 79 (75, 82) | 82 (78, 85) |
| *Between group difference* | 0.2 (-6, 6) | 6 (2, 11) | 7 (3, 11) | 7 (3, 12) | 6 (1, 11) | 7 (2, 13) | 6 (1, 10) | 6 (1, 11) | 6 (1, 12) |
| **Heart rate, beats/minute** |  |  |  |  |  |  |  |  |  |
| Glucocorticoid | 74 (69, 79) | 67 (63, 72) | 66 (61, 70) | 67 (62, 71) | 68 (63, 73) | 75 (70, 80) | 78 (73, 83) | 77 (72, 82) | 81 (76, 86) |
| Placebo | 76 (71, 81) | 69 (64, 73) | 70 (65, 74) | 70 (65, 75) | 70 (65, 75) | 77 (72, 82) | 80 (74, 85) | 80 (75, 85) | 82 (77, 87) |
| *Between group difference* | -2 (-9, 5) | -2 (-8, 5) | -4 (-11, 2) | -4 (-11, 2) | -3 (-9, 3) | -3 (-10, 4) | -3 (-10, 5) | -3 (-10, 4) | -2 (-10, 6) |
| **Vasoactive-inotropic score** |  |  |  |  |  |  |  |  |  |
| Glucocorticoid | 0.6 (0.3, 1.1) | 4.8 (2.8, 6.9) | 6.2 (3.8, 8.6) | 6.5 (3.9, 9.1) | 5.2 (3.0, 7.5) | 3.0 (1.2, 4.7) | 2.4 (0.9, 4.0) | 1.8 (0.4, 3.2) | 0.6 (-0.1, 1.3) |
| Placebo | 0.5 (0.007, 1.0) | 7.6 (5.0, 10.1) | 10.0 (7.0, 13.0) | 10.4 (7.1, 13.6) | 9.5 (6.5, 12.5) | 5.5 (3.1, 8.0) | 3.9 (1.9, 5.9) | 3.1 (1.3, 4.9) | 2.3 (0.8, 3.7) |
| *Between group difference* | 0.1 (-0.7, 0.8) | -2.7 (-6.0, 0.5) | -3.8 (-7.7, 0.1) | -3.9 (-8.1, 0.3) | -4.2 (-8.0, -0.5) | -2.6 (-5.6, 0.4) | -1.5 (-4.0, 1.1) | -1.3 (-3.6, 1.0) | -1.7 (-3.3, -0.1) |
| **VIS/MAP-ratio** |  |  |  |  |  |  |  |  |  |
| Glucocorticoid | 0.008 (0.0002, 0.02) | 0.07 (0.04, 0.10) | 0.08 (0.05, 0.12) | 0.09 (0.05, 0.12) | 0.07 (0.04, 0.10) | 0.04 (0.01, 0.06) | 0.04 (0.01, 0.06) | 0.03 (0.007, 0.05) | 0.008 (0.0002, 0.02) |
| Placebo | 0.007 (-0.0002, 0.01) | 0.11 (0.08, 0.15) | 0.15 (0.11, 0.20) | 0.18 (0.13, 0.23) | 0.17 (0.12, 0.23) | 0.10 (0.06, 0.14) | 0.07 (0.03, 0.10) | 0.06 (0.03, 0.09) | 0.04 (0.02, 0.07) |
| *Between group difference* | 0.001 (-0.01, 0.01) | -0.04 (-0.09, 0.003) | -0.07 (-0.13, -0.01) | -0.09 (-0.16, -0.03) | -0.10 (-0.16, -0.04) | -0.06 (-0.11, -0.01) | -0.03 (-0.07, 0.01) | -0.03 (-0.07, 0.01) | -0.03 (-0.06, -0.01) |
| MAP, mean arterial pressure; VIS, vasoactive-inotropic score  Treatment-by-time effects presented as estimated marginal means with 95% confidence intervals for each treatment group and the between group difference with 95% confidence intervals | | | | | | | | | |

**Supplementary table 3. Patient characteristics of patients with- or without PAC inserted**

|  | **PAC groups*** | |  |
| --- | --- | --- | --- |
|  | **No PAC**, N = 60 | **PAC**, N = 54 | **p-value** |
| **Before inclusion** |  |  |  |
| ***Demographic characteristics*** |  |  |  |
| Age, years, median (IQR) | 71 (62, 76) | 62 (54, 69) | <0.001 |
| Male, n (%) | 47 (78%) | 43 (80%) | 0.9 |
| Ischemic heart disease, n (%) | 15 (25%) | 9 (17%) | 0.4 |
| Heart failure, n (%) | 16 (27%) | 8 (15%) | 0.2 |
| Atrial fibrillation, n (%) | 9 (15%) | 9 (17%) | 0.7 |
| ***Prehospital variables*** |  |  |  |
| Witnessed arrest, n (%) | 52 (87%) | 46 (85%) | 0.8 |
| Bystander CPR, n (%) | 51 (85%) | 48 (89%) | 0.5 |
| Epinephrine administered, n (%) | 38 (63%) | 29 (54%) | 0.3 |
| Amiodarone administered, n (%) | 23 (38%) | 18 (33%) | 0.6 |
| Time to ROSC, minutes, median (IQR) | 16 (13, 20) | 17 (12, 21) | 0.8 |
| Post-resuscitation ECG rhythm, n (%) |  |  | 0.7 |
| *Sinus rhythm* | 45 (75%) | 39 (72%) |  |
| *Atrial fibrillation* | 12 (20%) | 10 (19%) |  |
| *Other** | 3 (5%) | 5 (9%) |  |
| Post-resuscitation ECG, signs of ischemia, n (%) |  |  | 0.07 |
| *STEMI* | 23 (38%) | 25 (46%) |  |
| *LBBB or RBBB* | 24 (40%) | 10 (19%) |  |
| *Unspecific ischemia†* | 3 (5%) | 6 (11%) |  |
| *No ischemia* | 10 (17%) | 13 (24%) |  |
|  |  |  |  |
| **After inclusion, in-hospital** |  |  |  |
| ***Hospital arrival characteristics*** |  |  |  |
| LVEF at arrival, %, median (IQR) | 40 (30, 45) | 40 (25, 50) | 0.8 |
| Lactate at arrival, mmol/L, median (IQR) | 5.1 (3.2, 7.2) | 5.0 (2.8, 6.6) | 0.5 |
| Cardiogenic shock at arrival, n (%) | 3 (5%) | 5 (9%) | 0.5 |
| Acute CAG, n (%) | 28 (47%) | 41 (76%) | 0.001 |
| Acute PCI, n (%) | 19 (68%) | 21 (52%) | 0.2 |
| ***During admission*** |  |  |  |
| ICU length of stay, days, median (IQR) | 3.3 (1.7, 6.4) | 5.6 (3.4, 7.4) | 0.02 |
| Ventilator, days, median (IQR) | 1.9 (0.7, 3.8) | 2.4 (1.8, 3.9) | 0.05 |
| Best LVEF in the ICU, %, median (IQR) | 45 (30, 50) | 40 (30, 50) | 0.7 |
| Day 1, SOFA cardiovascular score, median (IQR) | 3.5 (3.0, 4.0) | 4.0 (3.0, 4.0) | 0.02 |
| Day 2, SOFA cardiovascular score, median (IQR) | 3.0 (3.0, 4.0) | 3.5 (3.0, 4.0) | 0.02 |
| Day 3, SOFA cardiovascular score, median (IQR) | 0.0 (0.0, 3.0) | 3.0 (0.0, 3.0) | 0.2 |
| Day 1, SOFA total score, median (IQR) | 12.0 (11.0, 13.0) | 12.0 (11.0, 13.5) | 0.14 |
| Day 2, SOFA total score, median (IQR) | 10.0 (8.0, 12.0) | 11.0 (10.0, 12.0) | 0.10 |
| Day 3, SOFA total score, median (IQR) | 7.0 (4.2, 9.5) | 8.0 (5.0, 10.0) | 0.6 |
| Death before hospital discharge, n (%) | 22 (37%) | 13 (24%) | 0.15 |
| Death from any cause at 180 days, n (%) | 24 (40%) | 13 (24%) | 0.07 |
| Active treatment group, n (%) | 29 (48%) | 27 (50%) | 0.9 |
| CAG, coronary angiography; CPR, cardiopulmonary resuscitation; ECG, electrocardiogram; ICU, intensive care unit; IQR, interquartile range; LBBB, left bundle branch block; LVEF, left ventricular ejection fraction; PCI, percutaneous coronary intervention; RBBB, right bundle branch block; ROSC, return of spontaneous circulation; SOFA, sequential organ failure assessment; STEMI, ST-elevation myocardial infarction  *PAC was only available for insertion at the site “Rigshospitalet”  †Including pace rhythm, nodal rhythm, and sinus bradycardia  ‡Non-specific ST-segment depression/elevation or T-wave changes | | | |

**Supplementary figure legends**

*Figure 1:* Neurological outcome decided by CPC and mRS scores a minimum of 180 days after out-of-hospital cardiac arrest

*Figure 2, A-D*: Biomarkers of cardiac injury; A) Troponin T (ng/L) according to randomization, depicted as estimated marginal means and 95% confidence intervals to each time point including the p-value for the treatment-by-time interaction, B) Troponin I (ng/L) according to randomization, depicted as estimated marginal means and 95% confidence intervals to each time point including the p-value for the treatment-by-time interaction, C) Creatine Kinase MB (ug/L) according to randomization, depicted as estimated marginal means and 95% confidence intervals to each time point including the p-value for the treatment-by-time interaction, D) NT-proBNP (pmol/L) according to randomization, depicted as estimated marginal means and 95% confidence intervals to each time point to demonstrate differences between treatment groups. The figure includes Troponin T and Creatine Kinase MB measurements for all patients admitted at the site “Rigshospitalet” (n=81), while only Troponin I was available at the other site “Gentofte Hospital” (n=33). NT-proBNP measurements were available at both sites with missing values from two patients (n=112).

**Supplementary figure 1**


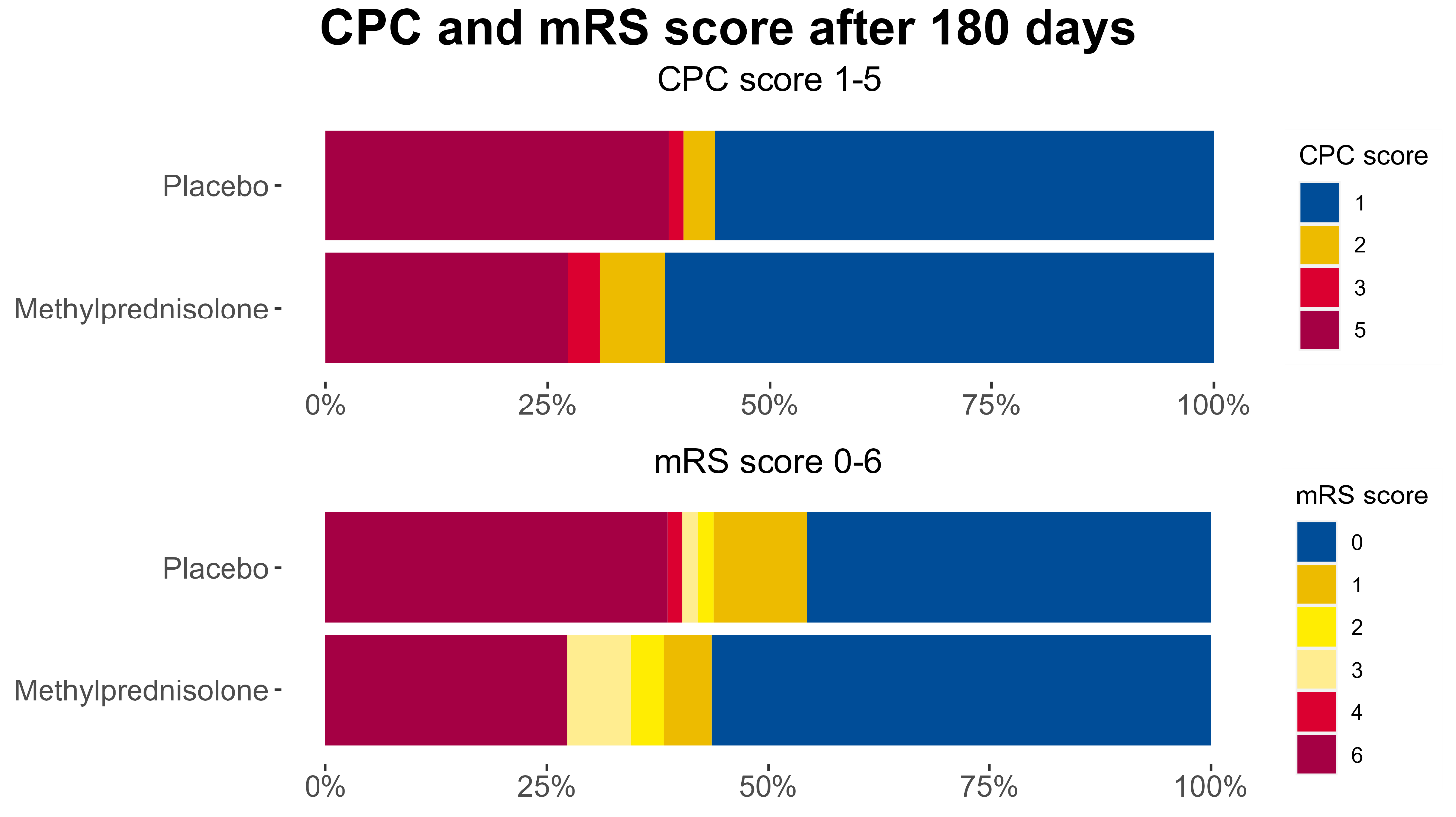


**Supplementary figure 2**

**
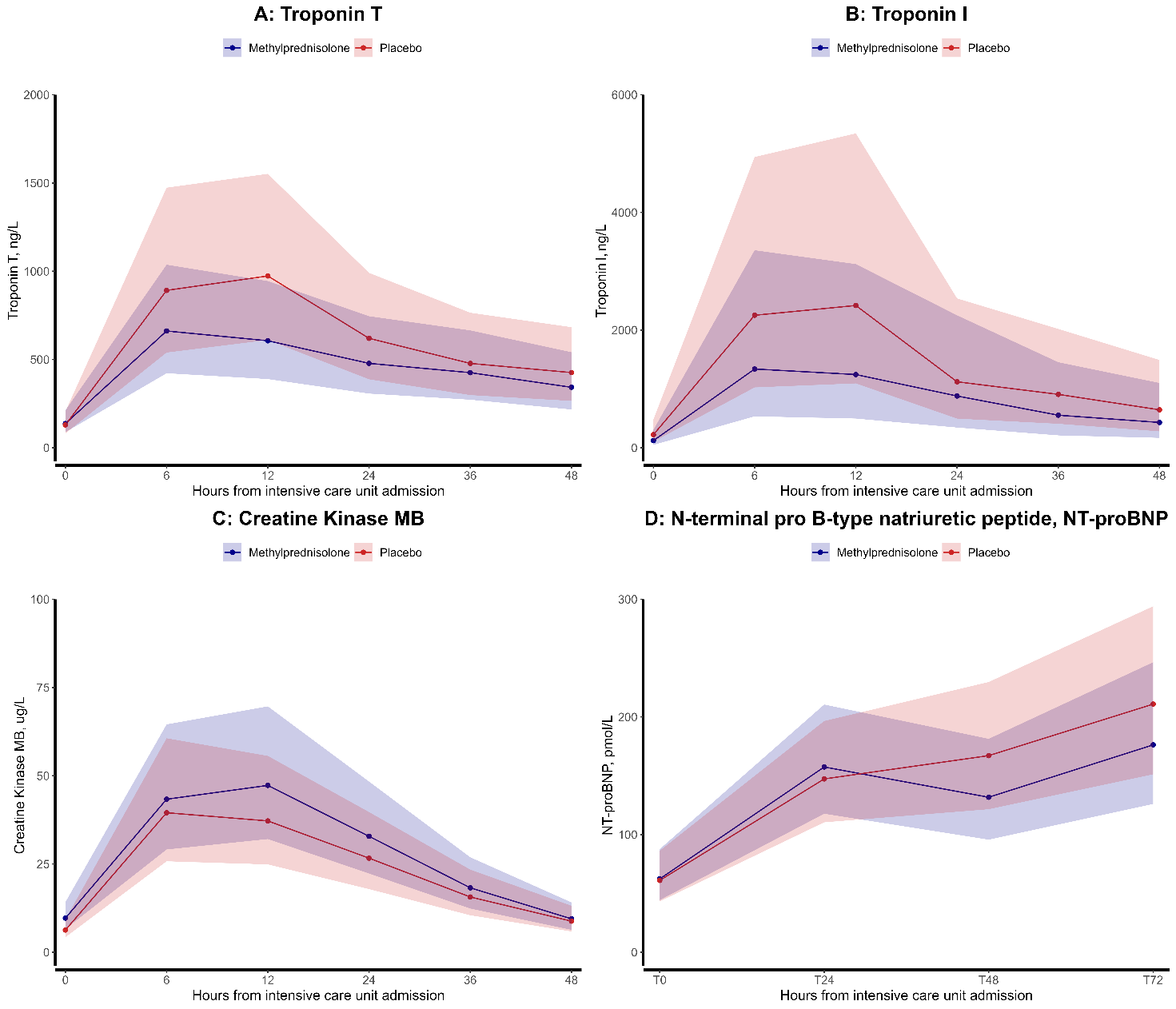
**
